# MRI Features of Stroke-Like Episodes in Mitochondrial Encephalomyopathy With Lactic Acidosis and Stroke-Like Episodes

**DOI:** 10.3389/fneur.2022.843386

**Published:** 2022-02-09

**Authors:** Weiqin Cheng, Yuting Zhang, Ling He

**Affiliations:** Ministry of Education Key Laboratory of Child Development and Disorders, Chongqing Key Laboratory of Pediatrics, National Clinical Research Center for Child Health and Disorders, China International Science and Technology Cooperation Base of Child Development and Critical Disorders, Department of Radiology, Children's Hospital of Chongqing Medical University, Chongqing, China

**Keywords:** mitochondrial myopathy, encephalopathy, lactic acidosis and stroke-like episodes (MELAS), stroke-like episodes, MRI, neuroimaging, application

## Abstract

Mitochondrial myopathy encephalopathy lactic acidosis and stroke-like episodes (MELAS) is an important cause of stroke-mimicking diseases that predominantly affect patients before 40 years of age. MELAS results from gene mutations in either mitochondrial DNA (mtDNA) or nuclear DNA (nDNA) responsible for the wide spectrum of clinical symptoms and imaging findings. Neurological manifestations can present with stroke-like episodes (the cardinal features of MELAS), epilepsy, cognitive and mental disorders, or recurrent headaches. Magnetic resonance imaging (MRI) is an important tool for detecting stroke-like lesions, accurate recognition of imaging findings is important in guiding clinical decision making in MELAS patients. With the development of neuroimaging technologies, MRI plays an increasingly important role in course monitoring and efficacy assessment of the disease. In this article, we provide an overview of the neuroimaging features and the application of novel MRI techniques in MELAS syndrome.

## Introduction

Mitochondrial encephalomyopathy with lactic acidosis and stroke-like episodes (MELAS) is a maternally inherited disorder caused by mitochondrial DNA (mtDNA) or nuclear DNA (nDNA) mutations in a diffuse multisystemic fashion. MELAS varies widely in the disease onset, symptoms, severity and prognosis. Its broad clinical presentation includes stroke-like episodes (SLEs), epilepsy, lactic acidemia, myopathy, hearing impairment, diabetes, cardiomyopathy and short stature (1, 2). The strong dependence of the central nervous system on oxidative metabolism predisposes to mitochondrial damage (3), and SLEs are the predominant features of MELAS (4). Typical neurological manifestations of SLEs are very similar to ischemic stroke in the acute phase. The diagnosis of MELAS is not difficult when the clinical and imaging findings are typical. However, due to the variability of the disease, diagnosis remains challenging and MELAS is easily misdiagnosed as cerebral infarction, viral encephalitis and other brain diseases, especially the first attack.

At present, with the wide application of new magnetic resonance imaging (MRI) techniques in the clinic, such as hydrogen proton magnetic resonance spectroscopy (1H-MRS), perfusion-weighted imaging (PWI) and arterial spin labeling (ASL), MRI has also become one of the effective methods for diagnosis of MELAS in addition to muscle biopsy and pathogenic gene testing. Clinicians have also gradually gained a deeper understanding of MELAS. In this paper, we review the conventional findings and the latest application of MRI in MELAS syndrome (Table 1).

**Table 1:** The neuroimaging features of stroke-like lesions in MRI.

	**Acute stage**	**No-acute stage**	
		**Sub-acute phase**	**Chronic stage**
T1WI	Hypointensity	Hyperintensity	Hypointensity
T2WI	Hyperintensity (bright thickened cortical band)	Hypointensity (black toenail sign)	Hyperintensity
T2FLAIR	Hyperintensity	Hypo/hyperintensity	
T1WI C+	Patchy/linear enhancement	No enhancement	
DWI	Hyperintensity	Normal	
ADC	Hypo/iso/hyperintensity	Normal	
MRS	An increased lactate peak	An increased lactate peak	
PWI/ASL	Hyperperfusion	Hypoperfusion	
MRA	Major vessels dilation/ normal/stenosis	Normal	
Characteristics	Lesions mainly distribute in the cerebral cortex and subcortex white matter with a predilection to the posterior brain, not limited to arterial territories and migratory		

*ADC, apparent diffusion coefficient; ASL, arterial spin labeling; DWI, diffusion-weighted imaging; MRA, magnetic resonance angiography; MRI, magnetic resonance imaging; MRS, magnetic resonance spectroscopy; PWI, perfusion-weighted imaging*.

## Clinical and Pathophysiological Features

MELAS is commonly associated with the m.3243A>G tRNALeu (UUR) mutation. Childhood and early adulthood are typically the age of onset with 65–76% of cases occurring at or before the age of 20, but disease onset can occur at any age (1, 4). Yatsuga et al. (5) found the juvenile of MELAS was associated with significantly higher mortality and a more rapid disease progression than the adult. Generally the earlier the clinical phenotypes appear, the severer the disease develops (6). SLEs, as one of the cardinal symptoms, classically present as acute hemianopia, hemiparesis, or cortical blindness. SLEs are usually recurrent and can lead to serious long-term consequences, such as neurodegeneration, cognitive impairment (7).

Energy deficiency can stimulate the proliferation of mitochondria of smooth muscle and small vascular endothelial cells at the same time. In MELAS, a variety of factors can lead to the lack of nitric oxide that can maintain the relaxation function of vascular smooth muscle. Both can cause microvascular blood perfusion damage, lead to stroke like attack and other complications (1). The basic neuropathological changes of MELAS were comprised of spongiform degeneration, neuronal cell loss, glial proliferation, and demyelination (8, 9).

At present, the diagnosis of MELAS is a comprehensive diagnostic criterion combining imaging findings, pathological examination, genetic testing, or muscle biopsy results with clinical manifestations. Among them, the discovery of pathogenic mutations in mtDNA or nDNA genes and typical pathological changes in mitochondrial myopathy by muscle biopsy is the “gold standard” for the diagnosis (10).

## Conventional Imaging Features

**Figure 1:**
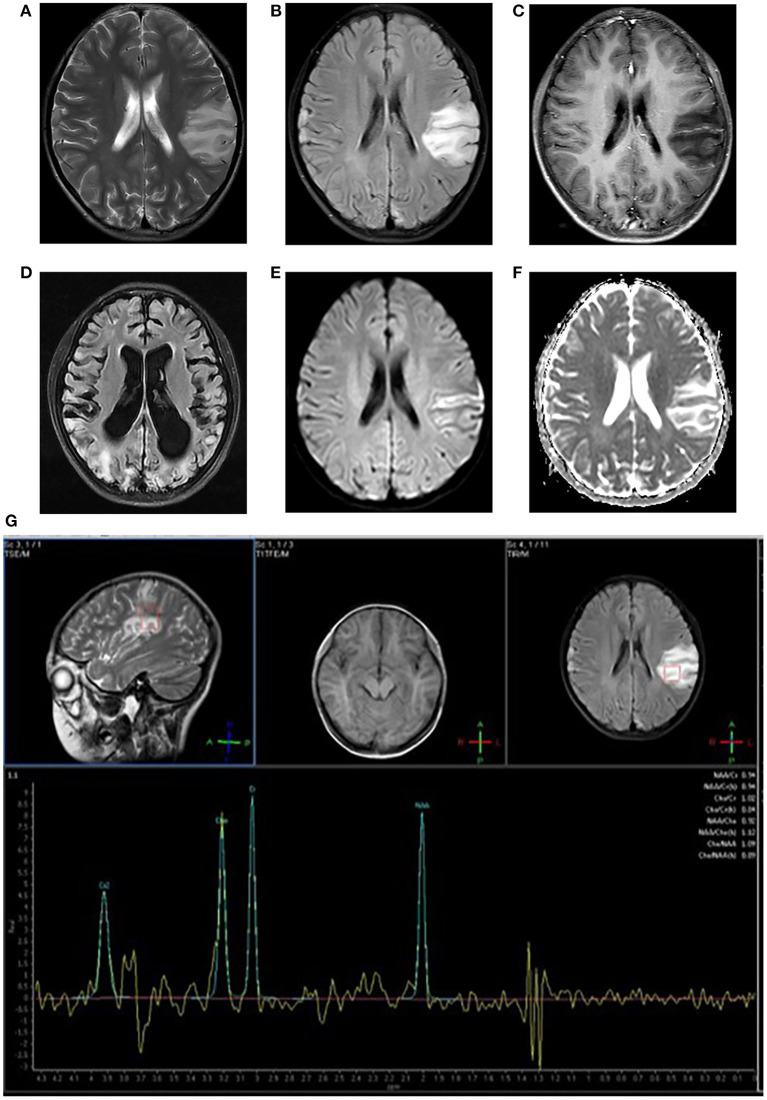
Neuroimaging for an 8-year-old girl with MELAS who presented with intermittent fever, vomiting, convulsions. **(A,B)** Axial T2WI and T2FLAIR imaging reveal multiple hyperintensities in bilateral frontal and parietal cortex and subcortical white matter, especially in the left side; **(C)** Axial post-contrast T1WI imaging reveals linear enhancement of the left lesions; **(D)** T2FLAIR image 3 years later demonstrates new migrating lesions of both cerebral hemispheres along with old lesions, accompanied by evolving encephalomalacia, atrophy; **(E)** DWI imaging demonstrates hyperintensities in gyriform pattern in the lesion areas; **(F)** ADC sequence shows iso/hyperintensities corresponding to DWI lesions; **(G)** MRS imaging shows decreased NAA/Cho ratio and a large lactate peak.

SLEs appear as stroke-like lesions (SLLs) on MRI. In acute SLEs, MRI findings include cortex swelling presenting with hyperintensity on T2WI and T2 FLAIR, named as “bright thickened cortical band” (Figures 1A,B). Part of cortical lesions show patchy or linear enhancement on T1-weighted postcontrast images (Figure 1C), due to local exudation or circulation disorders caused by the breakdown of the blood-brain barrier and increased regional cerebral blood flow in the affected areas (11). In the sub-acute phase, SLLs may develop gyriform hyperintensity on T1WI and hypointensity on T2WI/T2FLAIR (“black toenail sign”) because of cortical laminar necrosis. Whitehead et al. (12) found that the black toenail sign was a common imaging feature in MELAS, and the extent of gyral necrosis correlated with disease duration. A recent study observed that a cortical linear cystic lesion was a characteristic MR finding in MELAS patients (13), and it was defined as showing a linear or dotted cerebrospinal fluid signal in the deep layer of the affected cortex, and an iso-intensity line covered its surface. In the chronic stage, the affected areas gradually evolve into cerebral encephalomalacia, gliosis, and atrophy over time (Figure 1D).

Typical SLLs in MELAS mainly distribute in the cerebral cortex and subcortex white matter with a predilection to the posterior brain, although the deep gray matter such as the thalamus may also be affected probably reflecting its high metabolic demand (14). Lesions in the parietal and occipital lobe were twice as many as those in the temporal lobe and 4 times as many as those in the frontal lobe (15). Tschampa et al. (16) reported that deep gray matter changes presented in the majority of m.3243A>G mutation carriers lacking SLEs. Cortical lesions are typically multiple and asymmetrical; however, more and more symmetric cases have been recognized (17, 18), Bhatia et al. (18) thought highly specific cortical symmetry should also raise the possibility of MELAS. SLLs frequently spread to the cortex of adjacent gyri in a migratory fashion over time, resulting in large regions of cortical involvement that are not limited to vascular territories (4, 15, 19). The migratory, increasing and decreasing pattern of SLLs on imaging is the main feature of MELAS.

## Funtional MRI Findings

### Diffusion-Weighted Imaging and Apparent Diffusion Coefficient

Stroke-like lesions (SLLs) always present high signals on DWI (Figure 1E). Earlier reports suggested that the ADC value of SLLs was normal or increased (reflecting vasogenic edema) (20, 21), but now most studies have found that the ADC signals alternately change or mix in different periods (18, 22–24) (Figure 1F). Stoquart-Elsankari et al. (24) speculated that the changes in ADC might be related to the different levels of impairment of mitochondrial energy transport, correlated with cellular dysfunction. Moderate cellular dysfunction with vasogenic edema results from mild energy failure, and irreversible cellular failure responsible for cytotoxic edema is caused by severe energy failure. Besides, Xu et al. (25) reported a pattern of acute SLLs that DWI hyperintensity with decreased ADC along cortical area and increased ADC in most affected subcortical white matter. After the acute phase, the ADC value can return to normal.

### 1H-MRS

MELAS is characterized by an increased lactate peak in the lesion area, accompanied by a decreased N-acetylaspartate peak on 1H-MRS (Figure 1G). However, the characters are not specific and could also be found in other diseases, such as infarction. A lactate peak on MRS reflects anaerobic metabolism. Some reports have shown that a lactate peak even occurs in the normal-appearing region (brain parenchyma or cerebrospinal fluid area) on MRS (26, 27), which is of greater clinical significance for the disease. However, lactate signal could only be detected in normal cerebrospinal fluid in about one-third of patients (28).

Abe et al. (29) found that a patient 48 h after SLEs, a lactate peak on MRS could be seen much before the changes in DWI sequence, suggesting that MRS may have the predictive ability in displaying early lesions. Previous studies indicated that MRS might be more sensitive for detecting MELAS-associated preclinical abnormalities compared to conventional MRI (30, 31). Moreover, the lactate level varies at different stages of the disease. The lactate peak was bigger during onset than in intermission (30). Lactate in the lateral ventricles increased over time, and high lactate was associated with increased mortality (32). Weiduschat et al. (33) reported that lactate and total choline levels were reliable biomarkers for predicting the risk of individual A3243G mutation carriers to develop the MELAS. In addition, a recent study demonstrated the lactate peaks and ratios of N-acetylaspartate to choline were significantly improved that corresponded with clinical improvement after L-arginine therapy (34). To sum up, MRS may be a useful imaging biomarker for early diagnosis, course monitoring, and efficacy evaluation of MELAS.

### PWI and ASL

Both PWI and ASL can reflect microscopic hemodynamic information of the brain and evaluate cerebral perfusion. And as a non-invasive technique, ASL provides new dimensions in the evaluation of cerebral perfusion. The general trend is hyperperfusion in the acute stage and hypoperfusion in the chronic phase of SLEs (35–38). Hyperperfusion might be caused by dilation of cerebral arteries and increased microvascular permeability in the lesion areas (39), and hypoperfusion could be associated with cerebral cytotoxic edema, cortical atrophy, and gliosis (40). Li et al. (41) identified focal hyperperfusion as an imaging hallmark in acute encephalopathy of MELAS. In addition, regional hyperperfusion was observed on ASL in the preclinical phase 3–5 months before the clinical onset of SLEs (40), similar results were also reported in two other teams (41, 42). These reports indicate that ASL has the potential for detecting latent SLLs and predicting the emergence of SLEs. Meanwhile, Rodan et al. (43) found that MELAS disease severity and mutation load were negatively correlated with interictal cerebrovascular reactivity and directly correlated with frontal cerebral blood flow on ASL, suggesting that these metrics could be used as non-invasive prognostic markers to stratify risk for SLEs.

### Magnetic Resonance Angiography

MRA has not been routinely performed in MELAS in the past, because major cerebral vessels were considered to be the target of mitochondrial metabolism defects in these patients. However, more and more studies have found major cerebral vessels dilation (39, 44, 45) or stenosis (46, 47) on MRA in MELAS in the acute and chronic stages of the disease. Gramegna et al. (48) found that the proportion of cerebral major vessels dilation and stenosis was 40 and 19%, respectively, on MRA, and the middle cerebral artery was the most commonly involved. Among them, 88% of dilation was related to the acute SLEs, whereas only a few cases of stenosis were symptomatic for SLEs. Most alterations related to SLEs in the major cerebral vessels could be normalized completely after resolution of symptoms. These studies demonstrated that MRA could detect alterations in major cerebral vessels in MELAS patients. Furthermore, vasodilation by MRA had occasionally been detected in patients up to 3–5 months before the onset of SLEs (40, 44), indicating that MRA might be used as a possible tool for future onset of SLEs in selected patients. In a word, the macrovascular changes on MRA and underlying pathophysiology mechanism of MELAS need to be further investigated in large cohort studies.

### Other Functional MRI

There are also a few reports on the application of other new MRI techniques in MELAS. Virtanen et al. (49) observed in patients that mild microstructural damage of white matter tracts with loss of directional organization and reduced brain volumes with diffusion tensor imaging. Mineral (calcium or iron) deposition in basal ganglia of MELAS could be demonstrated by susceptibility-weighted imaging (50). Furthermore, studies on monitoring the disease status and evaluating drug efficacy by blood oxygenation level dependent function magnetic resonance imaging (bold-fMRI) have also been reported. Wang et al. (51) reported that MELAS patients, particularly those at the acute stage, exhibited topological reorganization of the whole-brain functional network based on resting-state fMRI. They also found that MELAS patients spent more time in a state with weaker connectivity and less time in states with stronger connectivity, and patients at the acute stage exhibited that global efficiency was markedly increased while local efficiency was decreased, compared to the controls and the patients at the chronic period (52). Additionally, Rodan et al. (53) demonstrated that MELAS patients' fMRI activation in response to visual cortex stimulus was significantly increased in primary visual striate cortex V1 and extrastriate regions V2 to V5 after L-arginine treatment with task fMRI.

## Differential Diagnosis

The diversity and complexity of clinical and radiological manifestations in patients with MELAS pose a challenge to the diagnosis. In imaging, the unilateral isolated cortical lesion is easily misdiagnosed as acute cerebral infarction, viral encephalitis, and low-grade glioma, etc., especially infarction. In acute SLLs, MRI differentiation of MELAS from other diseases mainly includes the following points: (1) lesions first involve the cortex and less deep white matter; (2) lesions commonly affect occipital and parietal lobes; (3) lesions are not limited to arterial territories and migrate over time; (4) a lactate peak appears in lesions, even in the normal-appearing region (brain parenchyma or cerebrospinal fluid area) on MRS, which is one of the indicators of diagnostic specificity; (5) lesions always present as hyperperfusion on PWI/ASL.

For acute ischemic stroke, patients are often accompanied by risk factors such as hypertension, diabetes, and hyperlipemia. The lesions of infarction are confined to the boundary of the vascular territories, and present hypoperfusion on PWI/ASL, effectively distinguishing it from MELAS. Chong et al. (54) discovered a new scoring criterion based on the vessel flow void sign and hyperintense vessel sign in T2FLAIR images was helpful to differentiate infarction from MELAS on conventional MRI, with sensitivity and specificity of 92.3 and 85.0%, respectively. Furthermore, cortical linear cystic lesions might help to distinguish the two diseases (13).

For viral encephalitis, patients may have a high fever, meningeal irritation with lymphocytic pleocytosis, and an elevated protein level on cerebrospinal fluid. On imaging, viral encephalitis usually involves the limbic system, such as the frontal orbital gyrus, hippocampus and temporal lobe, rather than the parietal, and temporal lobe. The diffusion restriction uninvolved the entire lesions might be an important differential diagnostic sign between them (55). Functional MRI techniques are also valuable for accurate diagnosis.

For low-grade glioma, patients often present with chronic onset. On imaging, the conventional MRI findings of low-grade glioma are sometimes similar to the single SLL, and MRS can help distinguish them. On MRS, low-grade glioma often presents with elevated choline peak and decreased N-acetylaspartate peak, but usually without an increased lactic acid peak, which is a characteristic of SLL.

## Conclusions

In conclusion, MELAS is a rare progressive neurodegenerative disorder involving multi-organs. MELAS has obvious clinical heterogeneity as the clinical manifestations of different patients or the same patients vary in different phases, which makes the diagnosis a little arduous sometimes. However, neuroimaging of MRI demonstrates characteristic patterns of MELAS patients, including cortex swelling with a predilection for the posterior brain regions, not limited to arterial territories, hyperperfusion, and elevated lactate peak in both affected and non-affected regions, which may be found concurrently with encephalomalacia and atrophy. The recognition of these imaging features signs facilitates screening and early diagnosis of MELAS. Meanwhile, the novel MRI approaches have provided new dimensions in the evaluation of the disease. Multimodal MRI has shown great potential in risk stratification, course monitoring, progression, and efficacy evaluation of MELAS, and also provides a reference for understanding its neuropathological mechanism.

## Author Contributions

WC collected the data and wrote the manuscript. YZ and LH performed roles of the conception of this review and substantively revised it. All authors contributed to the article and approved the final manuscript.

## Funding

This study was supported by grants from the joint Chongqing Health Commission and Chongqing Science and Technology Bureau Medical Research Project (No: 2020FYYX128) and Chongqing Science and Technology Bureau, Technology Foresight and System Innovation Project (No: cstc2021jsyj-yzysbAX0019).

## Conflict of Interest

The authors declare that the research was conducted in the absence of any commercial or financial relationships that could be construed as a potential conflict of interest.

## Publisher's Note

All claims expressed in this article are solely those of the authors and do not necessarily represent those of their affiliated organizations, or those of the publisher, the editors and the reviewers. Any product that may be evaluated in this article, or claim that may be made by its manufacturer, is not guaranteed or endorsed by the publisher.
